# ROOTS - Circular policies for changing the biowaste system

**DOI:** 10.12688/openreseurope.15507.1

**Published:** 2023-05-16

**Authors:** Miguel Ángel Suárez, Elisa Gambuzzi, José M. Soriano Disla, Gemma Castejón, Giulio Poggiaroni, James Ling

**Affiliations:** 1 CETENMA, Cartagena, Spain; 2 European Biomass Industry Association, Brussels, Belgium; 3 Greenovate! Europe, Brussels, Belgium

**Keywords:** biowaste, recycling, waste, by-products, biopesticides, insects, animal feed, protein, single cell protein, investment, circular economy, circular bioeconomy, circular cities and regions

## Abstract

The circular economy has a huge potential to make our societies more sustainable and decarbonised, with a reduced impact on the planet’s resources. The deployment of innovative solutions in the field of urban biowaste valorisation and reuse is still hindered by numerous bottlenecks, such as technological readiness, funding and financing tools availability, quality and quantity of biowaste and regulatory barriers. The European Green Deal and associated legislative initiatives provide the opportunity to overcome the last ones.

To promote innovative solutions for the European circular bioeconomy and help to overcome the barriers for the deployment of a circular bioeconomy, five Horizon 2020 projects working on biowaste valorisation have teamed up. This joint initiative is named ROOTS - circulaR pOlicies for changing the biOwasTe System. The projects HOOP, VALUEWASTE, SCALIBUR, WaysTUP! and CITYLOOPS are piloting new solutions to transform urban biowaste (food waste and green waste) and wastewater into valuable products like feed, fertilisers, bioplastics, biopesticides, proteins and bioethanol. They use different processes and technologies, but they all rely on high levels of recycling/upcycling and propose valorisation solutions relevant to the uptake of a truly circular bioeconomy.

As a result of the work performed and experience acquired, a number of bottlenecks have been identified, on the following topics: biowaste prevention, recycling targets and treatment plants, waste and by-products, biopesticides, insects for animal feed, single cell protein, citizen behaviour, investment needs.

For each identified bottleneck, this open letter proposes specifically 1) policy recommendations for each level of governance, and 2) information about solutions, good practices and concrete experiences from the participating projects.

## Disclaimer

The views expressed in this article are those of the author(s). Publication in Open Research Europe does not imply endorsement of the European Commission.

## Introduction

The circular economy has a huge potential to make our societies more sustainable and decarbonised, with a reduced impact on the planet’s resources. The European Union (EU) has made a significant commitment to this model and several initiatives and projects have been launched since the approval of the first Circular Economy package (2015).

As 34% of European municipal waste is organic, valorisation of biowaste is a key tenet of a circular economy ( EEA, 2020). Indeed, the EU Bioeconomy Strategy (2018) sees cities becoming major circular bioeconomy hubs, where biowaste is a feedstock for safe and sustainable biobased products. Changes in the EU waste legislation are expected to lead to more quality biowaste becoming available for use in biorefineries from 2024.

The deployment of innovative solutions in the field of urban biowaste valorisation and reuse is still hindered by numerous bottlenecks, such as technological readiness, funding and financing tools availability, quality and quantity of biowaste and regulatory barriers. The European Green Deal and associated legislative initiatives provide the opportunity to overcome the last ones. This open letter offers a set of concrete recommendations for policy makers together with information about solutions, good practices and concrete experiences.

## The ROOTS Initiative

To promote innovative solutions for the European circular bioeconomy and help to overcome the barriers for the deployment of a circular bioeconomy, five Horizon 2020 projects working on biowaste valorisation have teamed up. This joint initiative is named ROOTS - circulaR pOlicies for changing the biOwasTe System.

The projects HOOP, VALUEWASTE, SCALIBUR, WaysTUP! and CITYLOOPS are piloting new solutions to transform urban biowaste (food waste and green waste) and wastewater into valuable products like feed, fertilisers, bioplastics, biopesticides, proteins and bioethanol. They use different processes and technologies, but they all rely on high levels of recycling/upcycling and propose valorisation solutions relevant to the uptake of a truly circular bioeconomy.

At a first stage, the ROOTS promoters shared their concerns on the regulatory barriers hindering the deployment of circular bioeconomy. The joint work resulted in the release of a first position paper in May 2021 ( HOOP *et al.*, 2021) discussing four policy issues and the related proposed recommendations. The promoting projects have advanced providing results and evidences. The ROOTS group has grown including one more project and the 25 European cities participating in the five projects provided feedbacks and shared their views. All the gathered knowledge was used to further develop the position paper.

As a result of the work performed and experience acquired, a number of bottlenecks have been identified. For each identified bottleneck, this open letter proposes specifically 1) policy recommendations for each level of governance, and 2) information about solutions, good practices and concrete experiences from the participating projects.

## Biowaste prevention

Municipal waste accounts for 27% of total waste generated in the EU (excluding mineral waste) ( EEA, 2022). According to the waste hierarchy, prevention is the management system with highest priority. The 2020 EU Circular Economy Action Plan aims to halve the quantity of municipal waste not recycled or prepared for reuse by 2030, while all EU Member States must recycle or prepare for reuse at least 60% of their municipal waste by 2030. As both targets are correlated, ambitious waste prevention actions will be key to reaching them ( Table 1). In the specific case of food waste, the Farm to Fork Strategy sets ambitious reduction targets, but it would be necessary to define targets for all sub-categories of biowaste: household food waste, green waste, HORECA waste, agri-food industrial waste, wastewater sludges. This recommendation arises from the fact that the generation mechanisms and chemical-physical characteristics of these streams are widely different, and so are the reduction potential and recycling/upcycling options.

**Table 1.** Recommendations for municipal waste prevention.

Recommendations	Level of governance
Include targets for specific biowaste streams prevention (household food waste, HORECA, agri-food industry, green waste) in the “ Commission’s guidance to prepare a waste prevention programme” ( EU c., 2012)	European
Include the request to report on the abovementioned targets in the “ Questionnaire for Member States reports on the implementation of Directive 2008/98/EC of the European Parliament and of the Council on waste” ( EU a., 2012).	European
Reduce green waste by fostering sustainable landscaping of green spaces	Local
Define a waste prevention plan with specific targets for the different streams of municipal waste	European, National and Local
Set compulsory food waste prevention targets and practices for large producers such as restaurants, hotels, supermarkets, hospitals	National and Regional

**Examples of solutions, good practices and concrete experiences**

- CITYLOOPS has developed food demand management models in Porto (Portugal) to minimise food waste generation.
- In the HOOP “Lighthouse” Kuopio (Finland), the waste management company Jätekukko encourages households to compost to reduce biowaste production. The unpublished report by the HOOP project (2022) of Kuopio urban metabolism reveals that they generate approximately 40% less biowaste than the average of the other HOOP Lighthouse Cities and Regions.

## Recycling targets and treatment plants

In 2019, the EU Member States generated more than 225 million tonnes of municipal solid waste, 34 % of which were biowaste ( Eurostat, 2023). According to the Waste Framework Directive (WFD, 2008/98/EC); EU, 2008 and the Directive 2018/851, ( EU a, 2018) EU countries need to collect biowaste separately or ensure recycling at source from the end of 2023 onwards. Despite there is no specific recycling target for biowaste at EU level, biowaste is key to achieve the EU target to recycle 65 % of municipal waste by 2035 ( Table 2).

**Table 2.** Recommendations for the improvement of recycling biowaste and biowaste treatment plants.

Recommendations	Level of governance
Establish recycling targets for biowaste. When possible, prioritise the implementation of door-to-door collection (see section Biowaste prevention).	European, National and Regional
Establish door-to-door collection schemes at least for commercial activities such as restaurants, hotels, supermarkets, groceries.	Regional and local
Establish mechanisms to incentivise the participation in the separate collection of biowaste ( ***e.g.*** pay-as- you-throw schemes)	Regional and local
Define the minimum percentage to be recovered in mechanical-biological treatment plants.	National and Regional

On the other hand, the absence of targets for specific biowaste streams hinders the deployment of biorefineries-based approaches, since their economic viability rely on a sufficient feedstock quantity and quality.

**Examples of solutions, good practices and concrete experiences**

- WaysTUP! is demonstrating the technological feasibility of the recovery of waste that currently cannot be re-introduced to the market and ends up in landfills or incinerators (for example, cellulosic rejections from wastewater treatment plants used to produce bioethanol and biosolvents, obtaining 115–130 litres of bioethanol out of one ton of rejections, according to the unpublished report from the WaysTUP! project (2022).
- In 2019 the HOOP Lighthouses of Münster (Germany) and Albano Laziale (Italy), that implemented the separate collection of biowaste more than 20 years ago, collected 87% and 94% respectively of the total generated biowaste, according to unpublished reports from Abfallwirtschaftsbetriebe Münster (2018) and Volsca Ambiente e Servizi S.p.A. (2022). In both cases, door-to-door collection is done. On the other hand, Greater Porto Area (Portugal) does not reach those numbers despite the efforts in separate collection and campaigns. LIPOR Waste Observatory data centre shows that data are related to the nature of the housing, as big apartment buildings find more difficulties in separate collection.

## Waste and by-products

Definitions of waste and by-products are included in the EU, 2008 and its amendment with Directive 2018/851 ( EU a, 2018). According to them, “waste is defined as any substance or object which the holder discards or intends or is required to discard”. However, a by-product (Art. 5.1 Directive 2008/98/CE) ( EU, 2008) is defined as *a substance or object resulting from a production process the primary aim of which is not the production of that substance or object is considered not to be waste, but to be a by-product if the following conditions are met:*

- *(a) further use of the substance or object is certain;*
- *(b) the substance or object can be used directly without any further processing other than normal industrial practice;*
- *(c) the substance or object is produced as an integral part of a production process; and*
- *(d) further use is lawful, i.e. the substance or object fulfils all relevant product, environmental and health protection requirements for the specific use and will not lead to overall adverse environmental or human health impacts.*

In terms of circular economy, the future use of waste is much more restricted than in the case of by-products. Moreover, this definition also implies that the status of by-product can be only considered for those coming from industrial processes or other economic activities ( *i.e.* agriculture) and not for those coming from separate collection from households. This, for example, affects used cooking oils from households, despite having similar characteristics to the oil generated in production facilities. As demonstrated in the project WaysTUP!, the restriction in the definition makes that specific separate collection from households or similar undergoes the waste management route regardless of the quality of the separately collected biowaste.

The End-of-Waste status is a bottleneck in the application of circular economy. The conditions for such evaluation are decentralised into the Member States, even at regional level, with unstandardized and potentially very long procedures. These administrative applications, act as a barrier for the entrepreneurship in circular economy ( *i.e.* biorefineries) and promote the use of industrial feedstocks only. Actually, the circular bioeconomy business models of many companies and potential start-ups depend on this End-of-Waste status of the feedstock, the lack of which forces companies to apply for waste management licenses to operate, that, again, can be a lengthy and obstructive process. The recognition of End-of-Waste status should be standardized at EU level for biowaste, similarly to what was done for scrap metals, glass and copper by EU Regulations 333/2011 ( EU b, 2011), 1179/2012 ( EU b, 2012) and 715/2013 ( EU b, 2013), respectively ( Table 3).

**Table 3.** Recommendations for usage of waste as a by-product in circular economy.

Recommendations	Level of governance
To establish the criteria required for End-of-Waste status for several kinds of urban biowaste ( ***i.e.*** food waste, green waste, used cooking oils), similarly to what achieved by EU Regulations 333/2011 ( EU b, 2011), 1179/2012 ( EU b, 2012) and 715/2013 ( EU b, 2013) for scrap metals, glass and copper, respectively. This should help to set an EU reference and to promote unlocking the End-of-Waste requirements on national (or regional) level. This can be run through the foreseen implementing acts in Directive 2018/851 ( EU a, 2018) (Art.6.2) or through more binding policy options (regulations, directives) if preferred. This should help to clarify and simplify the End-of-Waste procedures, especially in Member States with lower degree of development of policies in the area of biowaste valorisation.	European
Create a Fast-Track for the obtention of waste management licences for actors upcycling or recycling biowaste, to promote the integration of these feedstocks in a circular process.	National, regional
Create specific categories for products coming from biowaste, with their own requirements, allowing for multiple reuse, aligned with the principles of the circular economy. This might be considered in case the bioproduct does not fulfil all the general requirements but still complies with functionality and safety.	European, national

**Examples of solutions, good practices and concrete experiences**

- The technology provider for the production of bioplastics from used cooking oils, involved in WaysTUP! and HOOP, demonstrates that it is easy to analyse a feedstock to evaluate whether it meets technical specifications for their biotechnology process.
- Within SCALIBUR, WaysTUP! and HOOP, hydrolysis and pyrolysis are employed to treat biowaste, which properties are significantly modified in the process, transforming it into either a nutrient/culture medium for bioprocesses or a bioproduct (biochar) for further use.
- Regulatory barriers were experienced when installing VALUEWASTE pilot plant in Murcia (Spain) waste treatment facility. Apart from the environmental authorisation for the pilot plant which took more time than expected, two VALUEWASTE partners operating the pilot were requested to obtain a waste management licence, which delayed the beginning of the pilot plant operations.
- As conveniently described in the Section Single cell protein (SCP), UNIBIO has demonstrated the safety/functionality of their product using biogas as feedstock.

## Biopesticides

Biopesticides are defined as “low risk” plant protection products, which implies “not containing substances of concern, being sufficiently active, and not causing unnecessary pain and suffering to vertebrates to be controlled”, according to Regulation 1107/2009 ( EU c, 2009). However, biopesticides have to face several barriers for their application as biocontrol products in terms of: durability, risk assessment, infrastructure requirements for their application, knowledge transfer from distributers to farmers, effective integration in plant disease management protocols, integrated and/or customised formulations, harmonisation and mutual recognition procedures for regulatory purposes and the uncertainty about the secure registration of new biopesticides.

Nevertheless, the European Commission following the Farm to Fork Strategy has revised the EU’s pesticide framework and set an EU-binding 50% chemical pesticide reduction target by 2030 but leave Member States free to set their own national targets. This support will help to speed up the market uptake of these products.

Nowadays, the lack of specific regulation for biopesticides means that these forms of biocontrol have not yet been able to live up to their full potential, as currently it takes around a decade to reach the market.

We propose the following recommendations ( Table 4) to mitigate this situation:

**Table 4.** Policy recommendations to facilitate the market uptake of biopesticides.

Recommendations	Level of governance
Create a simplified specific regulatory framework based on scientific data coming from research activities and projects allowing for an easier commercialisation of biopesticides. This regulatory feature should consider the specific features of biopesticides in contrast to chemical pesticides, and act accordingly.	European, National and Regional
Elaborate guidance documents that will enable the EU farmers to have access to alternative treatments through biopesticides already in the market and update them regularly; enhance, promote and prioritise the use of biopesticides ***versus*** the chemical ones; integrate them in the Integrated Management Plan and in the Common Agricultural Policy and update them regularly; identify and quantify the environmental, social and economic benefits of using biopesticides ***versus*** their chemically-derived counterparts; facilitate the application and registration requirements in the EU including flexibility to new products.	European
Update the regulation/restriction/ban in those cases where the use of chemical pesticides has shown a clear negative effect on environment and human health and favour and promote the use of biopesticides as their direct substitutes.	European, National and Regional

**Examples of solutions, good practices and concrete experiences**

•    By means of two different processes based on submerged and solid-state fermentation of the separately collected urban biowaste, successfully implemented and validated at pilot scale, SCALIBUR produced *Bacillus thuringiensis* (Bt) ( *var. kurstaki* and *var. israelensis*, respectively) derived biopesticides. The final product is being tested (insecticide activity) in order to quantify its effect against a concrete pest. (Bt) Biopesticides are the most widely explored and commercially successful microbial insecticides. However, the traditional production process is based on the use of defined synthetic media and first-generation carbon sources. The approach of SCALIBUR’s project is based on the use of renewable sources (urban biowaste). The bioconversion of urban biowaste into biopesticides has many important considerations: (1) low-cost feedstock that increases market competitiveness of biopesticides over chemical counterparts; (2) generation of environmentally friendly bioproduct; (3) minimization of solid waste, thus promotion of circular economy; and (4) reduction of non-renewable resources use.

## Insects for animal feed

Insects are a great source of proteins and using biowaste to grow and feed insects could unlock several economic opportunities. However, the uptake of circular insect breeding for nutritional purposes faces obstacles like the prohibition to feed insects with biowaste, the limitation of species that can be fed with insects, and the spare number of insect species allowed for human and animal nutrition purposes.

The projects of ROOTS are developing value-chains based on insect-rearing for feed production. Until 2021, the use of insects for feed purposes was approved only for aquaculture. However, since the second half of 2021, insect protein is allowed for pig and poultry feed. This is in line with objectives of the Farm to Fork strategy, aiming to make livestock farming more sustainable and seek alternative feed materials. Insect-protein could be the answer to this challenge.

As discussed, the main challenge comes from the feedstock used for feeding insects since it is not allowed to use neither separately collected urban biowaste (tested in VALUEWASTE and WaysTUP!), nor digestate from anaerobic digestion of separately collected urban biowaste (tested in VALUEWASTE). This is stated in Regulation 767/2009 Annex III Chapter 1.6 for urban solid waste ( EU a, 2009). This Regulation (Annex III Chapter 1.6 for urban solid waste) makes an exception with catering waste (HORECA waste, SCALIBUR), which according to Regulation 142/2011 ( EU a, 2011) (Annex I 22) implementing the Regulation 1069/2009 ( EU b, 2009) (about animal by-products and derived products not intended for human consumption) is defined as “all waste food, including used cooking oil originating in restaurants, catering facilities and kitchens, including central kitchens and household kitchens.” This category also appears in the Regulation 2017/1017 ( EU a, 2017) amending Regulation (EU) No 68/2013 ( EU a, 2013) on the Catalogue of feed materials as catering reflux (Category 9.9.1 in Catalogue of Feed), defined as “All waste food containing material of animal origin including used cooking oil originating in restaurants, catering facilities and kitchens, including central kitchens and household kitchens”. However, despite being present in the Catalogue, this feedstock, which can be equivalent to HORECA waste (SCALIBUR), is not allowed for feeding insects to be used later in animal feed (Regulation 1069/2009 ( EU b, 2009), Art.11b: “The following uses of animal by-products and derived products shall be prohibited: (b) the feeding of farmed animals other than fur animals with catering waste or feed material containing or derived from catering waste”).

ROOTS pledges to favour the uptake of insect-based animal feed by bringing down all the remaining regulatory impediments and further enlarge its use to more species. Recommendations made on “Waste and by-products” will also help to overcome this particular challenge ( Table 5).

**Table 5.** List of policy recommendations to facilitate the market uptake of animal feeds of insectile origin.

Recommendations	Level of governance
Revision of Regulation 767/2009 ( EU a, 2009) on the placing on the market and use of feed (Annex III Chapter 1.6) and 1069/2009 ( EU b, 2009) in relation to article 11b provided that either the separately collected urban biowaste or the catering waste comply with one of these requirements: • Do not contain materials of animal origin as a result of Member State, Regional or local specific waste regulations • The biowaste feedstock fulfils the requirements to ensure that the insect feed complies with the technical, environmental and safety requirements, as well as with the requirements for management systems to demonstrate compliance with the criteria, including for quality control and self-monitoring, and accreditation, where appropriate. This would be in line with the End-of-Waste criterion (Art.6) from Directive 2018/851 ( EU a, 2018) amending Directive 2008/98/CE ( EU, 2008)	European
Ban on animal products in separate collection ( ***i.e.*:** adapting the separate collection to the existing regulations aimed at food safety)	National, regional, local
Focus food safety research programmes on promising biowaste upcycling technology for biowaste, with the aim to fund research projects aimed at demonstrating that the current regulations can be modified, fostering the upcycling of more biowaste fractions ( ***e.g.*** documenting prion content in the value chain of insects)	European
Increase the number of insect species for animal nutrition.	European

**Examples of solutions, good practices and concrete experiences**

•    Entomo Agroindustrial fed black soldier fly larvae with separately collected urban biowaste and digestate, while other VALUEWASTE partners tested the derived bioproduct safety. Preliminary results on the experimental *in vitro* models with human hepatic and intestinal cells are positive and indicative that it is possible to obtain safe compounds from this valorisation line. However, further research is needed to improve the selection and categorization of biowaste, in order to be able to establish the requirements that different types of biowaste must meet in order to be included in the food chain.

•    In the WaysTUP! project, the University of Alicante is testing the functionality of black soldier fly larvae as poultry feed, evaluating the quality of the produced meat. At the same time, vegetable agriculture by-products and source separated animal by-products are being tested (fish, coffee, meat) as feedstock for the larvae. Larvae meal has similar properties as conventional soybean meal but being more sustainable.

•    One of HOOP project developers, Invertapro, is farming yellow mealworm to be employed as source of protein in aquaculture feed, farm animal feed and pet food. To avoid regulatory barriers on the marketability of the bioproduct, insects are fed with agri-food by-products instead of biowaste.

## Single cell protein

Single cell protein (SCP) comes from unicellular microorganisms, like bacteria and microalgae, and can be produced in a circular way by employing treated biowaste and/or biogas as part of the feeding and microorganism’s growing media. More specifically, a circular growing media includes *i)* hydrolysed biomass (including biowaste) that can be used to produce culture broths, *ii)* biomethane ( *i.e.*: the methane produced by anaerobic digestion of biowaste), the nutrient for methanotrophic bacteria and *iii)* carbon dioxide from biogas, to grow microalgae.

The main challenge lies in the raw materials/feedstock used to produce the growing media. As in the case of Insects for animal feed, barriers are found in Regulation 767/2009 ( EU a, 2009), affecting the use of separately collected urban biowaste (VALUEWASTE, HOOP) and Regulation 1069/2009 ( EU b, 2009), affecting catering waste (HOOP). In this case the interpretation of the status of this SCP is more difficult for two reasons:

1.    It is not clear whether the SCP obtained from these growing media can be considered as derived products or not. Even though hydrolysis ( *Reduction of molecular size by appropriate treatment with water and either heat/pressure, enzymes or acid/alkali*) and fermentation ( *Process in which micro-organisms such as bacteria, fungi or yeasts either are produced or used on materials to modify their chemical composition or properties*) appear in the glossary of processes in Regulation 2017/1017 ( EU a, 2017) amending Regulation (EU) No 68/2013 ( EU a, 2013) on the Catalogue of feed materials (Annex, Part B), it is not clear that the SCP can be considered a derived product, as the hydrolysate or the biogas are used as culture media.

2.    Regulation 1069/2009 ( EU b, 2009) (Art 5) does not mention any end-point criteria (from which the Regulation is not applicable anymore) for those derived products intended for feed of farmed animals (Art 31), although it does for derived products intended for other applications (Articles 32, 33, 35 and 36).

In both cases (methanotrophic bacteria and microalgae) the SCP is not obtained directly from the waste, but it is transformed into an intermediate (biogas, hydrolysate) used for growing the microorganisms. Therefore, it is not clear whether circular SCP for both animal and human nutrition can be commercialized in EU due to regulation restrictions, even if the biowaste-derived feeding media have, as described above, very different characteristics from untreated biowaste.

We propose the recommendations in order to improve the legislation regarding SCP listed in Table 6.

**Table 6.** List of legislation recommendations for the market uptake of single cell protein.

Recommendations	Level of governance
Revision of Regulation 1069/2009 ( EU b, 2009) (Art.5) in order to set or to allow the conditions for setting (for instance through Regulation 142/2011 ( EU a, 2011)) the end-point criteria related to the production of feed for farmed animals, so that it is clear when the bioproduct stops being a derived from animal by-product.	European
Revision of Regulation 142/2011 ( EU a, 2011) (Annex IV, Chapter IV Section 3, 1b) to include the use as culture media for microorganisms for the hydrolysates of Category 3 catering waste and similar (separate collection of biowaste from households), including also those microorganisms used for farmed animal feed	European
Revision of Regulation 142/2011 ( EU a, 2011) (Annex X, Chapter III) to include single cell protein as fish feed.	European
Ban on animal products in separate collection	National, regional, local

**Examples of solutions, good practices and concrete experiences**

- Greentech Innovators, one of HOOP project developers, produces microalgae for aquaculture feed from the hydrolysate from HORECA waste.
- At UNIBIO facilities, SCP is produced from methanotrophic bacteria and biogas coming from anaerobic digestion of separately collected urban biowaste. The product has been tested for functionality and safety. In terms of safety, and given the good results for some samples, there are reasons to be optimistic about the future. However, further research is needed on the fine-tuning of the biological process and on designing analysis and quality control strategies for the production of safe high value methanotrophic bacteria products. In terms of functionality, the addition of Uniprotein+ to food matrices resulted in beneficial nutritional values across all the tested matrices. In another functional/safety test with fish, results show that the replacement of fishmeal in commercial diets for rainbow trout by SCP produced by UNIBIO is a feasible and sound nutritional/health strategy in feed formulation.

## The behavioural issue

Citizens play a crucial role to enable biowaste valorisation routes towards high added value bioproducts. Indeed, most valorisation technologies rely on high quality biowaste feedstocks (low impurity content), while the availability of sufficient quantities represents an economic driver for the deployment of valorisation businesses. Citizens shall be well aware of how they can contribute to a fruitful separate collection scheme ( Table 7). The quality drastically depends on their (and the HORECA sector’s) ability to properly separate the biowaste from the rest of solid waste, in order to minimize the content of impurities or non-organic materials. The quantity requirement shall not be meant to throw as much biomass as possible, but to separate the non-avoidable fraction in the dedicated waste stream. In summary, poor biowaste separation is the result of careless sorting, low knowledge/interest about proper sorting, and lack of (adequate) systems to separately collect the urban biowaste or incentive/punitive systems. This can be addressed by policy tools.

**Table 7.** List of recommendation to improve the behavioural aspect.

Recommendations	Level of Governance
The EU and Member States should ensure the systematic application of penalization and/or reward systems at local level, to encourage citizens to properly sort biowaste. Actually, local governments, *i.e.*: the owners and responsible of generated waste, are often hesitant to promulgate ordinances that imply unpopular measures like economic penalization of citizens uncomplying with proper separate collection, because these measures are not legal imperatives.	European, national
Regarding the communication campaigns, resources are usually provided by the waste management companies, main beneficiaries of revenues associated to high-quality and quantity separately collected fractions. According to the EC Green Best Practice Community ( EU b, 2017), excellence case studies suggest to assess awareness-raising effectiveness according to a short list of indicators. One of them is to devote a proper budget to awareness-raising activities. • Private companies: Tender requirements for the selection of waste collection companies should include the obligation to invest in communication and awareness raising campaigns. (Local governance) • Public companies: Obligation to comply with specific standards for selected indicators (Regional governance).	Local, regional

One of the behavioural components related to biowaste sorting/consumption of bio-based products, is related to social acceptance. The acceptance of circular bio-based products is not discussed in this paper for three reasons: *i)* from VALUEWASTE experience ( VALUEWASTE D8.4, 2022), citizens are favourable to circular bioproducts; *ii*) no policy barriers are found and, finally, iii) to boost the presence and acceptance of circular bioproducts, scientific ( *i.e.*: evidence of safety) and marketing tools are more significant than policy tools.

**Examples of solutions, good practices and concrete experiences**

According to the experience of ROOTS projects, best performances in terms of separate collection are observed where bins can be associated to specific users. This strategy allows the conjoined application of reward and punishment mechanisms, that maximize the results in terms of quantity and quality of separate collection. As exemplified below, ROOTS partners that count on the highest quality and rate of separately collected biowaste adopt this strategy. The case of Murcia (Spain) is exposed as exemplary in communication campaign to start the separate collection from scratch.

•    In Albano Laziale (Italy), involved in both SCALIBUR and HOOP projects, the impurities in separately collected urban biowaste from household is very low (2–5% w/w). They adopted door-to-door collection with bar code bins and perform continuous information campaigns on how and why to separate waste. Slight reduction of waste management fees and recent application of the PAYT fee, always coupled with control and penalization (fines) of uncomplying citizens. An unpublished report from Volsca Ambiente Servizi e S.p.A (2022) found that after the implementation of the PAYT scheme, the percentage of putrescible material in separately collected biowaste increased from 86.27% (2019) to 90.22% (2022), revealing an improvement in sorting behaviour.

•    The separate collection of household biowaste in HOOP Lighthouse Münster (Germany) also counts on very low impurity levels (2–5% w/w). Actions such as door-to-door collection with bar code bins or continuous information campaigns have been carried out. AWM (public waste management company) created an educational trail on the closed landfill and counts on a department of education for sustainable development. AWM launched in 2017 the four-phases Aktion Biotonne campaign, an information campaign combined with the inspection of the quality of bio-bins of each household biowaste. If the quality found in the bins does not comply with standards, the citizens get a yellow card with instructions for better sorting. If the problem continues, the bio-bin is removed and household throws biowaste in the mixed-waste bin, with consequent application of the highest annual fee. With Aktion Biotonne, the impurities decreased from 3.5% in mid-2017 to 1.9% in early-2018, according to an unpublished report from the Institute for Infrastructure, Water, Resources and Environment (2018), indicating that the information campaign had an effect on the separation behaviour.

•    In Murcia (Spain), a city involved in both VALUEWASTE and HOOP projects, awareness campaigns were the first step to start the implementation of the separate collection of urban biowaste. Door-to-door information campaigns were carried out by the so-called “bio-patrols” ( *i.e.* dedicated staff whose mission is to interact with biowaste providers, usually in a face-to-face mode). In Murcia, the waste management company (Prezero) and Murcia City Council carried out specific campaigns to 1) explain citizens and biowaste large producers (mainly food markets) about the new separate collection system, 2) raise awareness on the high value products that can be obtained from biowaste. These campaigns supported the pilot experience implementation of the separate collection of urban biowaste that went from February 2020 until April 2022. During this experience, the average amount of improper biowaste found on citizens open bins went from 4.9% at the beginning, to 8.1% by the end, according to an unpublished report from the VALUEWASTE project (2022). This decrease in quality is related with increased participation ( *i.e.* higher quantity) and relaxation of educational campaigns focused on biowaste quality, which were affected by COVID-19. Thus, it is recommended to maintain active campaigns at all times on the subject of biowaste quality. Biowaste quality from large producers (average of 3% of improper waste), was better than the one reported for citizens.

•    Within the SCALIBUR project, an unpublished report from Las Dehesas methanisation plant, Madrid (2021) shows that Madrid (Spain) has increased the quality of the separately collected urban biowaste from 74.75% in 2019 to 80.73% in 2021 thanks to the continuous ongoing social awareness campaigns and engagement activities run by the city Council (“Acierta con la orgánica”, “cuando reciclo yo acierto” or “con erre de”), campaigns that have integrated the Biowaste Clubs organised within SCALIBUR and other social awareness activities, like webinars (“Sustainable Trends and Opportunities of the Retail Sector in Spain”) or the “Local Champions in Madrid”, who are local organizations in Madrid that have the power to influence a positive change in the community and consumers in general through the diffusion of practices related to circular economy and resource efficiency.

•    The VALUEWASTE consortium published the CEN Workshop Agreement on “Key factors for the successful implementation of urban biowaste selective collection schemes” ( CEN-CENELEC, 2022)

## Investment needs

Following the recommendations discussed above, the regulatory framework can be updated to overcome current and future challenges meeting specific needs. There is no doubt that a more favourable regulatory framework can facilitate the change, but in order to make the circular economy paradigm a reality, there is a need to address non-policy barriers. Probably, the most important one is that related to unlocking investment, either public or private. From this perspective, Europe is making a huge effort in mobilizing capital to foster the uptake of sustainable economic activities through funding and financing schemes, acting as a worldwide reference for the transition to a sustainable economy.

Thus, ROOTS partners suggest focusing on the following investment-related mechanisms in a way to speed up the fostering of circular bioeconomy:

•    Horizon Europe research and innovation funding programme to allocate resources to specifically deploy biowaste waste-to-product or waste-to-energy valorisation.

•    LIFE research and innovation funding programme to the scale-up and deployment of the aforementioned innovative solutions

•    Ensure the inclusion of circular bioeconomy criteria among the technical screening criteria for objective *4. Transition to a Circular Economy* in the EU Taxonomy, currently under development.

## The road ahead

The ROOTS group wants to play an important role in achieving a more sustainable society through circular biowaste valorisation schemes that comply with safety and health standards. We gathered to provide the perspective of Horizon 2020 projects and speak out loud to concretely contribute to transform and improve our society. For this reason, we must combine the development of new innovative solutions with the necessary dialogue with policy makers on regulatory barriers. Policy makers should pay more attention to the results arising from the hundreds of EU funded projects which constantly provide results, field experiences and best practices.

The ROOTS group will continue to operate, despite the conclusion of some of the organizing projects. Others will join in the future and the stakeholder community built in the past two years shall carry on the proposals presented here. 

Find out more about the ROOTS projects and reported public deliverables and activities on their respective websites:

- cityloops.eu
- hoopproject.eu
- scalibur.eu
- valuewaste.eu
- waystup.eu

## Ethics and consent

Ethical approval and consent were not required

## Data Availability

No data are associated with this article.
