# Supplementary material for: ROOTS - Circular policies for changing the biowaste system
Source: Open Res Eur. 2023 May 16;3:78. [Version 1] doi: 10.12688/openreseurope.15507.1 (PMC10446026; doi:10.12688/openreseurope.15507.1)
Supplement: Peer review history

# Peer review: Reviewer response for version 1

**DOI:** 10.21956/openreseurope.16764.r33863

**Published:** 2023-08-22

**Authors:** Benyamin Khoshnevisan

**Affiliations:** 1 Department of Green Technology, University of Southern Denmark, Odense, Denmark

# The deployment of innovative solutions in the field of urban biowaste valorisation and reuse is still hindered by numerous bottlenecks, such as technological readiness, funding and financing tools availability, quality and quantity of biowaste and regulatory barriers.

- Social aspects are better also included here. For instance, the social acceptance of the final products.

# EU state members currently have different sorting and source separation strategies. Could it be one aspect to align source sorting and source separation strategies?

 # In the HOOP “Lighthouse” Kuopio (Finland), the waste management company Jätekukko encourages households to compost to reduce biowaste production.

- I would not call it biowaste generation prevention. In such conditions, biowaste is generated. The only difference is its management strategy. Since the waste is generated and not prevented, I would argue that home composting is not the most circular and sustainable option at some points.

# Examples of solutions, good practices and concrete experiences.

- I would suggest adding one or two more examples here if possible.

# Waste and by-products

- I would also suggest the term Residuals or Resource residuals instead of waste. Once they are used as feedstock within the bioeconomy concept they are not waste any more but a resource.

# Single cell protein, bullet 1:

- Methane oxidizing bacteria and Hydrogen oxidizing bacteria are two types of SCP. The former grows on methane and the latter grows on hydrogen and carbon dioxide. While methane and carbon dioxide are supplied from biogas, they also need nitrogen to grow. Some studies show that nitrogen can be safely remove from biogas slurries, urine, digestate to grow SCP (e.g. see Khoshnevisan *et al.*, 2020 ^1^). Such strategy can ensure safety of final product.

Where applicable, are recommendations and next steps explained clearly for others to follow? (Please consider whether others in the research community would be able to implement guidelines or recommendations and/or constructively engage in the debate)

Partly

Does the article adequately reference differing views and opinions?

Yes

Are all factual statements correct, and are statements and arguments made adequately supported by citations?

Yes

Is the rationale for the Open Letter provided in sufficient detail? (Please consider whether existing challenges in the field are outlined clearly and whether the purpose of the letter is explained)

Yes

Is the Open Letter written in accessible language? (Please consider whether all subject-specific terms, concepts and abbreviations are explained)

Yes

Reviewer Expertise:

Waste management; Biowaste biorefiney; Sustainability assessment

I confirm that I have read this submission and believe that I have an appropriate level of expertise to confirm that it is of an acceptable scientific standard.

## References

[1] : Coupling electrochemical ammonia extraction and cultivation of methane oxidizing bacteria for production of microbial protein. *J Environ Manage* .2020;265: 10.1016/j.jenvman.2020.110560 110560 10.1016/j.jenvman.2020.110560 32421560

# Peer review: Reviewer response for version 1

**DOI:** 10.21956/openreseurope.16764.r33424

**Published:** 2023-07-28

**Authors:** Vinay Kumar

**Affiliations:** 1 Department of Community Medicine, Saveetha Institute of Medical and Technical Sciences, Chennai, India

The article entitled “ROOTS - Circular policies for changing the biowaste system” explores and explain about the policies related to biowaste system. The manuscript discusses the ROOTS initiatives, biowaste prevention, recycling targets, treatment plants, waste and by products, biopesticides, insects for animal feed and single cell proteins.

 The manuscript has been structured in a well manner. At the same time, it explains the key problems associated with the biowaste policies. In addition, it addresses the major regulations already available and their amendment recommendations. The article has been written good English syntax and is error free. In my opinion it will be a great asset for readers and researchers associated with biowaste management. The article is approved from my side, and it can be accepted for indexing.

Where applicable, are recommendations and next steps explained clearly for others to follow? (Please consider whether others in the research community would be able to implement guidelines or recommendations and/or constructively engage in the debate)

Yes

Does the article adequately reference differing views and opinions?

Yes

Are all factual statements correct, and are statements and arguments made adequately supported by citations?

Yes

Is the rationale for the Open Letter provided in sufficient detail? (Please consider whether existing challenges in the field are outlined clearly and whether the purpose of the letter is explained)

Yes

Is the Open Letter written in accessible language? (Please consider whether all subject-specific terms, concepts and abbreviations are explained)

Yes

Reviewer Expertise:

NA

I confirm that I have read this submission and believe that I have an appropriate level of expertise to confirm that it is of an acceptable scientific standard.
